# Snake scent gland secretions repel and induce contact toxicity in ants

**DOI:** 10.1007/s00114-025-01990-4

**Published:** 2025-05-20

**Authors:** Paul J. Weldon, Robert K. Vander Meer

**Affiliations:** 1 Smithsonian Conservation Biology Institute, National Zoological Park, 1500 Remount Road, Front Royal, VA 22630 USA; 2 Imported Fire Ant and Household Insects Research Unit, USDA, Center for Medical, Agricultural, and Veterinary Entomology, 1600 SW 23rd Drive, Gainesville, FL 32608 USA

**Keywords:** Ant repellent/toxicant, Formicinae, Myrmicinae, Serpentes, Snake defenses

## Abstract

Embedded in the tail base of all snakes is a pair of scent glands from which typically foul-smelling secretions are expelled when snakes are disturbed. The tendency of predatory ants to avoid snake cloacal fluids, and the abundance and structural diversity of potentially insecticidal carboxylic acids identified in scent gland secretions (SGS), prompted speculation that SGS function to deter ants. We examined the deterrent properties of the SGS of the Middle American burrowing python (*Loxocemus bicolor*) in fumigation, repellency, and contact-toxicity behavioral assays against workers of the red imported fire ant (*Solenopsis invicta*) and a species of carpenter ant (*Camponotus floridanus*), thus representing the two major ant sub-families, Myrmicinae and Formicinae, respectively. We also examined responses by *S. invicta* to the SGS of representative booid, pythonid, colubrine, elapinine, and crotaline snakes. None of the SGS samples affected the two ant species in fumigation tests. However, in repellency bioassays, ants given a choice between a droplet of water or sugar water versus a diluted droplet of SGS overwhelmingly avoided the latter, typically exhibiting rapid antennation from within a few mm, then retreating. Pure or diluted SGS applied directly to ants induced a high percentage of paralysis and death. Some treated ants exhibited symptoms of contact toxicosis but recovered within a 4-h observational period. Our results and reports of the responses of predatory ants to the Texas blindsnake (*Rena dulcis*) point to the scent glands as an ancient and widespread source of ant deterrents.

## Introduction

Embedded in the tail base of all snakes is a pair of holocrine glands known as scent glands. These organs, which are synapomorphic for Serpentes (Bellairs 1950; Whiting 1969), expel typically foul-smelling secretions through two duct openings at the posterolateral margin of the cloaca when snakes are disturbed (e.g., Greene 1987). Here, we report on scent gland secretions (SGS) as ant deterrents, various species of which prey upon or target snakes defensively in biting or stinging attacks (Savage 1849; Retenmeyer 1963; Gehlbach et al. 1968; Schneirla 1971; Webb and Shine 1993; Sazima 2015).

Studies of the defensive properties of SGS focused initially on the Texas blindsnake (*Rena dulcis*, Leptotyphlopidae: Epictinae). This snake, as with other scolecophidians (‘worm snakes’), enters ant and termite nests to feed on larvae and pupae (e.g., Greene 1997). When attacked by ants, *R. dulcis* coils into a tight ball and writhes, smearing itself with discharged cloacal fluids containing SGS (Gehlbach et al. 1968; Watkins et al. 1969). In laboratory tests, these discharged fluids were shown to repel three species of predatory ants – *Labidus coecus*, *Neivamyrmex nigresens*, and *Solenopsis geminata* – all of which preferentially resided on a half of a test arena treated with ethanol rather than on the side treated with an ethanol extract of the cloacal fluids of *R. dulcis* (Watkins et al. 1969). The repellency of the cloacal fluids of *R. dulcis* was attributed to carboxylic acids in its SGS (Blum et al. 1971).

An analysis of the SGS of the Middle American burrowing python (*Loxocemus bicolor*, Loxocemidae), a fossorial denizen of moist to dry forests from Mexico to Costa Rica, revealed an abundance of carboxylic acids and lesser amounts of alcohols and glycerol monoalkyl ethers (Schulze et al. 2017). Over 100 C_13_ – C_24_ carboxylic acids, including straight chain and mono-, di-, and trimethyl-branched compounds with up to three double bonds, were identified in the SGS of this snake. The structural diversity of these acids was hypothesized to reflect their activity as deterrents of ants and other leaf-litter arthropods.

We examined the effects of the SGS of *L. bicolor* and those of representative booid, pythonid, colubrine, elapid, and crotaline snakes on the red imported fire ant (*Solenopsis invicta*, Formicidae: Myrmicinae) in fumigation, repellency, and contact-toxicity behavioral assays. *Solenopsis invicta* is indigenous to northern Argentina (Calderra et al. 2008) but was introduced into the United States in the 1930’s and later, from there, into Australia, Taiwan, and China (Ascunce et al. 2011). It is a soil dwelling, opportunistic omnivore, infamous for inflicting painful stings on humans, livestock, and other animals. We also tested the SGS of *L. bicolor* against the carpenter ant, *Camponotus floridanus* (Formicidae: Formicinae), a chiefly insectivorous ant that ranges from North Carolina to Florida and west to Mississippi.

Phylogenetic studies date the most recent common ancestor of modern ants to the Early Cretaceous period, between 145 and 110 million years ago (Borowiec et al. 2020). The two ant species in our study represent the myrmicine and formicine lineages, the former of which is older, having originated ca. 99 million years ago (Ward et al. 2014). Snakes also arose in the Early Cretaceous period. Snakes used as SGS donors in our study represent diverse alethinophidian (‘typical snake’) lineages thought to have arisen during the Mid-Cretaceous period (Rage and Werner 1999).

## Materials and methods

### Source of ants used in bioassays

Mature, queenright, monogyne colonies of *S. invicta* were collected from the Gainesville, Florida vicinity and maintained at the USDA, Imported Fire Ant and Household Insects facility, Gainesville, Florida. Major workers were used in fumigation and contact-toxicity bioassays; minor and media workers were used in repellency bioassays. Fire ant colonies produce a continuous range of worker sizes with no distinct castes, but they are classified as minor, media, and major based on size (Tschinkel 2006).

*Camponotus floridanus* workers (300–400 individuals) were obtained from the Entomology and Nematology Department of the University of Florida, derived from a *C. floridanus* colony reared from a newly mated queen collected in July 2015. They were maintained in a 32 × 22 × 8 cm plastic tray, the upper inner walls of which were coated with Fluon® to keep ants from escaping. Two plastic Petri dishes with lids (diameter = 9 cm; height = 15 mm), placed 9 cm apart at opposite corners of the tray, had a floor consisting of Castone®, which was kept moist to humidify the Petri dish. The Petri dish lids had a hole in the center that allowed ants to move in and out.

All ants had access to adult crickets and 10% sugar water up to the time of testing for the fumigation and contact-toxicity bioassays. *Solenopsis invicta* and *C. floridanus* used in repellency tests were deprived of food and water for 16 and 48 h, respectively.

### Snake secretion collection

SGS were obtained from 19 adult male, female, and unsexed *L. bicolor* (TL (snake length) = 76—110 cm). All SGS collections entailed restraining snakes while applying manual pressure to the tail base. The expressed secretions were directed into 20-ml glass scintillation vials with foil lined caps (Wheaton, DWK986546, MilliporeSigma, St. Louis, MO). Some samples of *L. bicolor* were pooled from up to five individuals, with a total of 8.2 g of SGS obtained (mean yield = 430 mg/snake). The SGS of all snakes were kept pure. SGS collection vials generally were transported on dry ice, weighed, then frozen (−18 °C). Prior to use in bioassays samples of SGS were refrigerated (3 °C) for 24 h. Snakes were obtained from a variety of sources—see Acknowledgement section.

*Solenopsis invicta* workers also were tested in bioassays with SGS from a female northern boa constrictor (*Boa imperator*, Boidae: Boinae) (TL = 1.5 m, SGS yield = 1.1 g), a non-venomous constricting species that inhabits rainforests from Mexico to northwestern Colombia; four (unsexed) ball pythons (*Python regius*, Boidae: Pythoninae) (TL = 1.1–1.4 m, pooled SGS yield = 740 mg), a constricting species that inhabits grasslands, shrublands, and forests of sub-Saharan Africa; a female unicolor cribo (*Drymarchon melanurus unicolor*, Colubridae: Colubrinae) (TL = 1.7 m, SGS yield = 830 mg), a non-venomous predator of small vertebrates that inhabits marshes, swamps, and riverbeds from Mexico to Central America; a female king cobra (*Ophiophagus hannah *sensu lato, Elapidae, TL = 2.6 m, SGS yield = 490 mg), a venomous, chiefly ophiophagous snake endemic to the jungles of southern and southeast Asia; and a male timber rattlesnake (*Crotalus horridus*, Viperidae: Crotalinae) (TL = 0.8 m, SGS yield = 570 mg), a venomous pit viper that inhabits forests in the United States from southern Minnesota to New England and south to east Texas and north Florida.

The SGS of *Cr. horridus* were transported as described for *L. bicolor*. The samples of *B. imperator*, *P. regius*, *D. m. unicolor*, and *O. hannah* were chilled on ice and tested for fumigant activity within 2 to 7 h of collection.

### Bioassays

#### Fumigation

The plastic caps (25 mm diameter) of 20-ml glass scintillation vials of the type used to collect SGS, and 2-ml plastic tubes (diameter = 9 mm, length = 45 mm) with screwcap, were modified and combined to create a fumigation apparatus. A hole (diameter = 12 mm) was cut in the center of a vial cap, and a hole (diameter = 9 mm) was cut in the top of the screw-cap. The screwcap was glued into the hole of the vial cap with its thread facing downward so that the plastic tube to which it attached projected into the vial when the cap was screwed on. A 10 × 14-mm-hole was cut in the sidewall of the tube. Both the top and sidewall openings of the tube were covered with a fine plastic mesh to allow volatiles to enter. The utility of our fumigation apparatus was confirmed in preliminary tests with several organic solvents, which immediately immobilized ants. All fumigation, repellency, and contact-toxicity bioassays were conducted at 21- 23 °C.

#### Solenopsis invicta

Samples of SGS used in our bioassays were first used in fumigation tests. Five *S. invicta* major workers were placed into the plastic tube and the tube was then affixed to its screw-cap. The tube with ants was inserted into a vial containing 1.5 g of SGS pooled from four adult *L. bicolor*. After 1 h of confinement, the cap was unscrewed from the vial and the screwcap on the tube was opened to release the ants into a Petri dish, where they were observed for 1 min. After each trial, the fumigation apparatus was washed with soapy water, rinsed with distilled water and then ethanol, and allowed to dry overnight. This procedure was repeated four times (N = 20).

The bioassay described above was conducted with SGS from *B. imperator* (1.1 g), four *P. regius* (740 mg), *D. m. unicolor* (830 mg), *O. hannah* (490 mg), and *Cr. horridus* (570 mg). SGS of the *B. imperator* and *D. m. unicolor* were evaluated within 2 h of collection. The bioassay was repeated four times for each snake species.

#### Camponotus floridanus

The SGS of *L. bicolor* (980 mg pooled from five individuals) were tested for fumigant activity with *C. floridanus*. This species produces formic acid for defense and readily releases this irritant when disturbed. We modified the experimental protocol for this species by confining one ant at a time in our apparatus for 1 h to avoid aggression stemming from overcrowding and overexposure to formic acid. The bioassay was repeated on 20 individual ants.

### Repellency

#### Solenopsis invicta

**Fig. 1:**
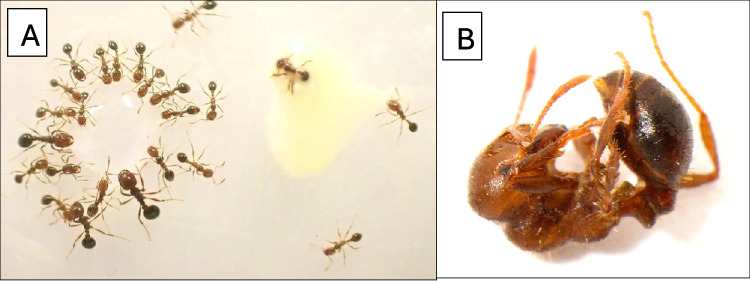
**A** left: *S. invicta* worker ants drinking from a water droplet; right: an SGS spiked water droplet, 2 ants walking away and one trying to get out of the droplet. **B** A dead, contorted *S. invicta* worker 4 h after receiving an SDS treatment on its gaster/petiole

Approximately 200 media and minor workers of *S. invicta* were confined in a 135 × 135 × 50-mm Fluon®-lined plastic bowl 16 h before testing. The repellency assay entailed pipetting a 300-μl droplet of distilled water onto one side of a 40 × 40-mm sheet of aluminum foil. A droplet of 200 μl of SGS was pipetted 15—25 mm away from the water droplet, and 100 μl of distilled water were added to it, thus equalizing the volume of the droplets. The ends of the foil sheet were upturned, and it was gently placed on the floor at the center of the bowl. The number of ants encircling (and presumably drinking from) each droplet was recorded at 2 min (see Fig. 1A).

The dark green SGS of *B. imperator* had a paste-like consistency that prevented it from being manipulated by micro-pipette. To prepare these SGS for our repellency assay, 30 μl of water were added to 30 mg of SGS and the mixture was stirred. For both trials, a 10-μl droplet of water was applied to a piece of aluminum foil and 20 μl of the diluted SGS were added to it by micro-pipette; a 30 μl droplet of distilled water was placed 20 mm away for the control, as in the tests with the other snakes.

#### Camponotus floridanus

The *C. floridanus* workers responded poorly to water droplets necessitating a change in protocol. A 0.5 M sucrose solution was prepared, which was a phagostimulant to *C. floridanus* workers. Worker ants were tested in their home-colony container. Forty μl of the 0.5 M sucrose solution and 20 μl of a 1 M sucrose solution, to which 20 μl of the SGS of *L. bicolor* were added, were placed 3 cm apart from each other on a 5 × 10 cm strip of aluminum foil. The strip was then placed in the middle of the container diagonally between the two Petri dish shelters.

The lid of their shelter was gently lifted, thus inducing ants to exit the Petri dish into the colony container. The position of the sugar versus sugar + secretion droplet on the aluminum foil strip, and the Petri dish opening for ants to explore the strip, were alternated with each trial. In contrast with *S. invicta*, where ants attending droplets were recorded at 2 min, the number of individual *C. floridanus* at each droplet was recorded each min for 6 min, hence this scoring method entailed repeated observations.

### Contact toxicity

#### Solenopsis invicta

To investigate the contact toxicity of SGS against *S. invicta*, thawed SGS was applied to major workers. The control was the application of distilled water. Each ant was held with feather forceps while 8 μl of SGS or distilled water was micro-pipetted onto the petiole and gaster. Ants were then placed individually on the inside of a Petri dish lid (diameter = 14.2 cm), usually with fluid clinging to them. The lid was then inverted over the bottom dish (diameter = 13.1 cm) and tapped, causing the ants to drop to the dish floor. The SGS from the *Boa imperator*, had a paste-like consistency, thus we modified our protocol by daubing ants with a ca. 10 mg droplet of SCS on the tip of a needle.

The condition of each ant was noted at 5 min and 4 h following the treatment application. Ants were deemed unaffected if they walked normally or did so after being prodded with a needle tip. Ants were considered to have exhibited symptoms of toxicosis if they jerked their body spasmodically or were inverted and flailed their antennae and/or legs. Ants were deemed dead if they were motionless and failed to respond to gentle prodding, or if they were moribund at the 4 h evaluation. Treated ants often assumed a contorted posture within a few minutes after the application of SGS (see Fig. 1B).

#### Camponotus floridanus

Preliminary tests with *C. floridanus* indicated that they readily succumb when topically treated with fluids, even water. We therefore modified our contact-toxicity bioassay by reducing the volume of fluid applied to these ants and limiting the anatomical site to which SGS were applied. Each ant was held with feather forceps while 5 μl of *L. bicolor* SGS or water were applied to the dorsal and/or ventral aspect of the gaster. Ants were then transferred to Petri dishes and observed as described for *S. invicta*.

## Statistical analyses

All graphs and statistical analyses were performed using GraphPad Prism 10.1.2. For analyses involving the toxic effects of the SGS, unpaired, two-tailed t-tests were used to compare the mean surviving workers in the treatment versus the mean surviving workers in the controls. All quantities are reported as the mean ± the standard error.

## Results

### Fumigation

All *S. invicta* and *C. floridanus* workers behaved normally following the hour-long confinement with SGS in the fumigation apparatus.

### Repellency

#### Solenopsis invicta

Workers of *S. invicta* were given a choice between droplets of water versus droplets of *L. bicolor* diluted SGS. After 2 min, workers encircled the water droplets. In contrast, ants typically approached the SGS-laden droplets, antennated them from within a few mm, and retreated (see Fig. 1A). However, two ants, one in each trial, walked into the SGS droplet and became immobile; these ants were not counted. Therefore, a total of 31 ants surrounded the water droplets, whereas no ants attended the SGS-laden water droplets.

*Solenopsis invicta* responded similarly to the SGS of the other snakes. At 2 min in both trials with *B. imperator*, 15 and 20 ants encircled the water droplets; however, none contacted the SGS-laden droplets [totals: 35 versus 0]. At 2 min in both trials with *P. regius*, 33 and 35 ants encircled the water droplets; 3 and 7 ants contacted the SGS-ladened droplets [totals: 68 versus 10]. At 2 min in both trials with *Drymarchon*, 10 and 19 ants encircled the water droplets; none contacted the SGS-laden droplets [totals: 29 versus 0]. At 2 min in both trials with *O. hannah*, 17 and 27 ants contacted the water droplets. Three ants contacted the SGS-laden droplet in one trial; none contacted it in the second trial [totals: 44 versus 3]. At 2 min in both trials with *C. horridus*, 11 and 29 ants encircled the water droplets. Three ants were immersed in the SGS droplet and appeared to be dead during one trial; none aggregated around the SGS-laden droplet in the other trial [totals: 40 versus 0]. For *P. regius*, the result with the greatest number of ants at the SGS sample (7) had 35 at the water. Simple chi-squared analysis with a total of 42 responding ants and a null hypothesis of 21 ants at the treatment and control gave Χ^2^ = 18.667, P < 0.0001. Therefore, by inference the results for all four snake species showed that significantly fewer ants responded to the aqueous SGS samples compared to aqueous controls.

#### Camponotus floridanus

In three bioassays, the number of *C. floridanus* workers at sugar water versus sugar water plus *L. bicolor* SGS were 17 to 1, 12 to 2, and 12 to 0 (totals: 41 versus 3). As with *S. invicta*, these results show significant repellent effects of the SGS. For the 12 to 2 replicates, Χ^2^ = 7.143, P < 0.0075, therefore, by inference all 3 bioassays showed that sugar water diluted SGS repelled *C. floridanus* workers.

### Contact toxicity

#### Solenopsis invicta

The SGS from *Loxocemus bicolor*, *Drymarchon melanurus unicolor*, *Crotalus horridus*, and *Boa imperator* snake species were evaluated for toxicity effects on *S. invicta* workers by applying a small amount of pure SGS to an individual ant’s petiole/gaster.

**Fig. 2:**
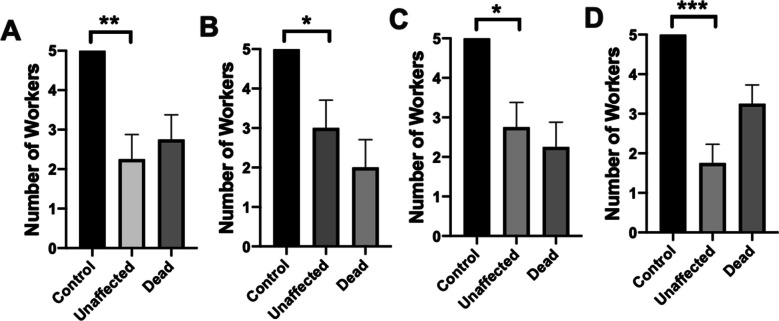
Each graph shows the results (mean + SE of 4 replicates) for controls, worker ants unaffected by the SGS treatment, and the number of dead worker ants 4 h after treatment (worker ants still moribund after 4 h after treatment were considered dead). **A** The SGS treatment was from *Loxocemus bicolor*. **B** The SGS treatment was from *Drymarchon melanurus unicolor*. **C** The SGS was from *Crotalus horridus*. **D** The SGS was from *Boa imperator*. The SGS from the four snake species showed a significant decrease in live workers compared to the control. * = *P* < 0.05; ** = *P* < 0.01; *** = *P* > 0.001

Treatment of *S. invicta* workers with the SGS from *Loxocemus bicolor* resulted in a significant decrease in functional workers (N = 4), Fig. 2A, unpaired two-tailed t test, t = 4.371; P = 0.0047. Results for the treatment of *S. invicta* workers with the SGS from *Drymarchon melanurus unicolor* is shown in Fig. 2B. There was a significant decrease in functional workers, unpaired two-tailed t test, t = 2.828; P = 0.0300. Figure 2C shows the results for treatment of workers with the SGS from *Crotalus horridus*, which resulted in a significant decrease in functional workers, unpaired two-tailed t test, t = 3.576; P = 0.0117. Treatment of workers with the SGS from *Boa imperator* resulted in a significant decrease in functional workers, Fig. 2D, unpaired two-tailed t test, t = 6.789; P = 0.0005.

#### Camponotus floridanus

Treatment of *Camponotus floridanus* with the SGS from *Loxocemus bicolor* resulted in a 20% decrease in functional workers. After 4 h one treated ant died from 3 of the 4 replicates, and in the 4 th replicate 1 worker was moribund through 4 h and was classified as dead. Since the 4 replicates had identical results a Wilcoxon matched pairs signed rank test was used to test for a significant difference. The exact 2-tailed P = 0.1250; therefore, the results for the control and treatments after 4 h were not significantly different.

## Discussion

The SGS of *R. dulcis* and other snakes have been examined as deterrents of predatory vertebrates in several studies. Experiments or anecdotal reports indicate that ophiophagous and other heterospecific snakes avoided zones of test arenas treated with discharged cloacal fluids (Watkins et al. 1969), and that American alligators (*Alligator mississippiensis*) (Weldon and McNease 1991) and carnivores (Price and LaPointe 1981; Wright and Weldon 1990) refused, reluctantly consumed, or disgorged SGS-treated food. As Weldon and Fagre (1989) pointed out, however, carnivorans evolved after the origin of snakes, hence scent glands as a source of defensive chemicals likely arose in response to earlier predators.

We examined the effects of SGS from a taxonomically diverse assemblage of snakes on two species of distantly related ants in fumigant, repellent, and contact-toxicity behavioral assays. These bioassays generally are used to assess insects’ responses to incapacitating vapors, feeding deterrents, and contact insecticides. A comparable battery of behavioral assays was used in a study of responses by ants, including *S. xyloni*, to the defensive secretions of heteropteran insects (Eliyahu et al. 2012).

Our fumigation assay failed to indicate that SGS vapors induce observable negative effects “e.g., physical impairment or death” in worker ants, despite the ample exposure they received to SGS volatiles. Ants in some experiments were exposed to freshly collected (within 2 h) SGS to maximize exposure to volatiles, but they nonetheless were unaffected.

The repellency of SGS extracts, on the other hand, was indicated in each of our feeding tests: ants congregated around or sequentially visited water or sugar-laden droplets overwhelmingly more than droplets containing SGS. Both ant species in our study often approached and closely antennated SGS-laden droplets, but generally retreated without contacting them. This observation suggests that ants detected and avoided volatile compounds emanating from the diluted SCS. These results and those of the fumigation experiments suggest that the detected compounds have low volatility and are in accord with Gehlbach et al.’s (1968) observation that ants avoided the side of a test arena treated with an extract of the cloacal fluids of *R. dulcis*.

Our results also indicate that SGS act as contact toxicants against ants, an activity observed indirectly in repellent bioassays and directly in contact-toxicity bioassays. In the repellent bioassays, ants became immobile and died if they breached the SGS-treated droplets. Bioassays that directly applied SGS from four snake species onto worker ants led to 49, 40, 45, and 65% mortality respectively (see Fig. 2). In a different system of reptilian chemical defense, lice exposed to the aldehyde-laden (fumigant) odor of the crested auklet (*Aethia cristatella*) also exhibited paralysis, with some individuals recovering (Douglas 2013).

Aside from the Gehlbach et al. (1968) report on the responses by captive *R. dulcis* to several species of predatory ants from Texas, detailed accounts of snake-ant encounters are scarce; hence, evidence implicating scent-gland discharge as a defense against ants is limited. Webb and Shine (1993) staged encounters between the Australian blindsnake, *Ramphotyphlops nigrescens*, and bulldog ants (*Myrmecia*) and observed that small to medium-sized blindsnakes discharged cloacal fluids only after being stung by large worker ants. These investigators failed, however, to observe that these fluids affected the ants. Whether the ants in Webb and Shine’s study contacted the snakes’ cloacal fluids, as appears to be necessary for SGS-induced toxicosis, is unclear.

Blum et al. (1971) posited that the repellence of ants by the cloacal fluids of *R. dulcis* is due to a series of C_12_ – C_20_ carboxylic acids that are dissolved in a glycoprotein matrix of this snake’s SGS. They pointed out that carboxylic acids generally are insecticidal and thus may deter ants. The contact toxicity of carboxylic acids has been demonstrated against a broad spectrum of insects, including hard-bodied taxa, e.g., coleopterans (Seigler and Popenoe 1924, 1925, Sims et al. 2014, Démare et al. 2018, Ren et al. 2019, Krzyżowski et al. 2020).

Aside from the abundance of carboxylic acids in *R. dulcis* and *L. bicolor*, they are the sole or predominant lipoidal components in the SGS of booid (Tolson 1987; Simpson et al. 1988, 1993), pythonid (Wood et al. 1995), colubrid (Oldak 1976; Wood et al. 1995), elapid (Weldon et al 1991), and viperid snakes (Razakov and Sadykov 1986; Weldon et al. 1992; Young et al. 1999). The insecticidal activities of carboxylic acids, and their ubiquity and abundance in SGS, are consistent with their contribution to snakes’ chemical defense against ants. Moreover, short-chain (C_2_ – C_5_) carboxylic acids, which occur widely in the SGS of alethinophidians (Wood et al. 1995; Weldon et al. 2022), are prime candidates to exert repellence. Bioassay-guided isolation of active components is needed to identify SGS components that act as ant deterrents.

Scent glands are synapomorphic in the Serpentes, thus they can be traced back to the earliest snake. The habitus of the first snakes, however, is controversial (Gower et al. 2021). Some molecular analyses indicate that modern scolecophidians represent an ancient phenotype inherited from the last common ancestor of snakes (Miralles et al. 2018). The inferred subterranean lifestyle of early snakes and the similarities of their skull morphology to that of some extant blindsnakes are consistent with their subsistence on a diet of brood from ant and termite nests. SGS may have permitted snakes to enter these nests. Whether or not this accurately characterizes the role of SGS for early snakes, several modern snakes, including colubrids and pythonids, enter ant and termite nests to feed, oviposit, and shelter (Riley et al. 1985; Holm 2008; Baer et al. 2009; Pisani 2009; Thompson and Thompson 2015). SGS may play an underappreciated role in allowing snakes access to these generally restrictive domains.

## Data Availability

Data will be made available on request.
